# Design of Concrete Mix Proportion Based on Particle Packing Voidage and Test Research on Compressive Strength and Elastic Modulus of Concrete

**DOI:** 10.3390/ma14030623

**Published:** 2021-01-29

**Authors:** Yun-Hong Cheng, Bao-Long Zhu, Si-Hui Yang, Bai-Qiang Tong

**Affiliations:** School of Resources and Civil Engineering, Northeastern University, Shenyang 110819, China; 1770779@stu.neu.edu.cn (B.-L.Z.); 1800975@stu.neu.edu.cn (S.-H.Y.); 1870821@stu.neu.edu.cn (B.-Q.T.)

**Keywords:** concrete, voidage, concrete mix design, compressive strength, elastic modulus

## Abstract

According to the basic principle of dense packing of particles, and considering the interaction between particles, a dense packing model of granular materials in concrete was proposed. During the establishment of this model, binary particle packing tests of crushed stone and sand were carried out. The fitting analysis of the test results determines the relationship between the particle size ratio and the remaining volume fraction of the particle packing, and then the actual void fraction of the particle packing was obtained, based on which the water–binder ratio was combined to determine the amount of various materials in the concrete. The proposed concrete mix design method was used to prepare concrete, and its compressive strength and elastic modulus were tested experimentally. The test results show that the aggregate volume fraction of the prepared concrete increased, and the workability of the concrete mixture with the appropriate amount of water reducing agent meets the design requirements. When the water–binder ratio was 0.42, 0.47, or 0.52, the compressive strength of the concrete increased compared with the control concrete, and the degree of improvement in compressive strength increased with the decrease in water–binder ratio; when the water-binder ratio was 0.42, 0.47, or 0.52, the static elastic modulus of the concrete increased compared with the control concrete, and the degree of improvement in elastic modulus also increased with the decrease in water–binder ratio. The elastic modulus and compressive strength of the prepared concrete have a positive correlation. Findings show that the concrete mix design method proposed by this research is feasible and advanced in a sense.

## 1. Introduction

The concrete mix ratio is the proportional relationship between the amount of each constituent material in concrete, and the performance of concrete is closely related to its mix ratio. There are different methods for designing mix ratios in different countries. The American ACI method is simple and easy, and the concrete mix ratio can be determined by looking up the relevant forms [1]. The British BRE method is similar to the ACI method in parameter selection, but more factors are taken into consideration in mix ratio design [2]. the French Dreux method considers the parameters in the mixture ratio design more accurately. The French de.larrad method is based on physical and mathematical models, so it is better in theory [3]. the Chinese JG55 method [4] is of semi-empirical design, and for the majority of China it is a universal and operable concrete mix design method.

Among the constituent materials of concrete, coarse aggregate, fine aggregate, and cementitious materials are all granular materials, and it can be said that concrete is a dense solid formed by filling these granular materials with each other [5]. According to the principles of particle packing and material science, the way in which granular materials are packed in concrete has a great impact on their macro-mechanical behavior; that is, the denser the particle packing, the smaller the voids, and the more contact points among particles, which theoretically leads to a higher strength of concrete [6]. Dense packing means that when particles are packed, the spaces between large particles are filled by smaller particles, and the spaces between smaller particles are then filled by smaller particles [7], so as to achieve maximum compactness. Therefore, the design of concrete mix based on densely packed particles has attracted the attention of researchers. Huang Zhao-long [8,9,10] proposed the “Dense Counter-fill Mix Proportion Design Method”, which firstly fills the fly ash in the sand, and then fills the best mixture of fly ash and sand in the crushed stone, thus finding the maximum unit weight of each solid material in the concrete through packing tests and then obtaining the minimum void ratio. Inspired by the “Dense Counter-fill Mix Proportion Design Method”, Wang Ling-ling [11] proposed the “Dense Positive-fill Mix Proportion Design Method”, which differs from the “Dense Counter-fill Mix Proportion Design Method” in that the material is filled in a different order; that is, sand is first filled into the crushed stone, and then fly ash is filled into the optimal mixture of sand and crushed stone. Wang Li-jiu [12] proposed the “Infilled Coefficient Mix Design of Concrete”, in which the infilled coefficient reflects the proportional relationship between the filler and the aggregate of the concrete; in concrete designed by this method, the cement consumption per cubic meter of concrete is reduced, and the fluidity and strength of the concrete are improved. Fu Pei-xing [13], on the basis of the characteristics of heterogeneous cement-based composites, pointed out that concrete is composed of four volumes: cement paste, air, sand, and stone; they proposed that the concrete mix ratio design should be based on these four volume ratios, in which the amount of sand and gravel is determined according to the principle of dense packing. Nan Su [14] proposed a mix design method for medium-strength flowing concrete with low cement content; this method determines the packing factor (aggregate content) first, and then binding paste is filled in the voids between aggregates to make concrete that has the desired workability and strength. In the method proposed by H.F. Campos [15], the proportions between fine materials and aggregates are based on particle packing techniques; his method was used for producing a kind of high-strength concrete that had been tested and proved to have a high compressive strength and high elastic modulus. In addition to the above review, there are many other related studies [16,17,18,19].

This research proposes a new method for designing thew concrete mix ratio. The method is based on the binary particle packing test of crushed stone and sand, and the relationship between the particle size ratio and the remaining volume fraction of particle packing is determined through the analysis of the test results. Furthermore, the actual voidage of the particle packing is obtained, and then the water–binder ratio is combined to determine the amount of various materials in the concrete. Then, the proposed concrete mix design method is used to prepare concrete, and its compressive strength and elastic modulus, the two most basic parameters in the design of concrete structures, are tested.

## 2. Particle Packing Model in Concrete

### 2.1. Basic Model of Particle Packing

Coarse aggregates, fine aggregates, and cementitious materials are granular materials in concrete. Considering the mutual filling of particles with different particle sizes, the particles are packed with the goal of achieving maximum compactness or minimum void ratio after the particles are mixed. The basic model expression for particle packing is as follows:

Coarse aggregate volume fraction:

(1) $$y_{1}=1-\varphi_{1}$$

Fine aggregate volume fraction:

(2) $$y_{2}=\varphi_{1}\cdot (1-\varphi_{2})$$

Volume fraction of cementitious material:

(3) $$y_{3}=\varphi_{1}\cdot \varphi_{2}\cdot (1-\varphi_{3})$$

Total volume fraction for particles packing:

(4) $$y_{3}=\varphi_{1}\cdot \varphi_{2}\cdot (1-\varphi_{3})$$

where, $\varphi_{1},\varphi_{2}\;\mathrm{and}\;\varphi_{3}$ are the voidage of coarse aggregate, fine aggregate, and cementitious material, respectively (%). In addition,

(5) $$\varphi_{i}=1-\frac{\rho_{0i}}{{\rho_{0i}}^{\prime }}$$

where, $\rho_{0i}\;\mathrm{and}\;{\rho_{0i}}^{\prime }$ are the apparent density and natural bulk density of each material, respectively, in kg/m^3^.

This packing model is proposed based on the theoretical dense packing between particles with different particle sizes; that is, fine aggregates are filled in the gaps of coarse aggregates, and cementitious materials are filled in the gaps of coarse and fine aggregates.

### 2.2. Interactions between Particles in the Packing System

When particles of different sizes are blended, they have a spatial influence on each other, described by the wedging effect [20]. When coarse particles are dominant, virtually all fine particles will fill into the voids between coarse particles. However, some isolated fine particles may be trapped in the narrow gaps between the coarse particles, rather than filling into the voids between the coarse particles. As a result, coarse particles are wedged apart, resulting in the formation of voids at the gaps between coarse particles. When fine particles are dominant, the coarse particles will disperse themselves into the sea of fine particles so that there is always a gap between adjacent coarse particles. However, the gap width may be unevenly distributed, and some gaps may be relatively small. Where the gap is too small to accommodate even one layer of fine particles, the layer of them within the gap cannot be a complete one, and there may be just one or two isolated fine particles within the gap, resulting in the formation of relatively large voids.

The wedging effect makes the actual voidage of the particle pack larger than the theoretical voidage of the particle pack, and this interaction between the particles is related to the particle size ratio between the particles [20].

### 2.3. Modification of the Basic Model of Particle Packing

#### 2.3.1. Particle Binary Packing Test

Particles with sizes of 22.75, 17.5, 12.75, 7.125, 3.555, 1.77, 0.89, 0.45, and 0.225 mm were selected, and each size was the arithmetic mean of the adjacent sieve sizes of the standard sieve for concrete sand and gravel specified in *Standard for Technical Requirements and Test Method of Sand and Crushed Stone (or Gravel) for Ordinary Concrete* (National standard of the People’s Republic of China, JGJ52-2006).

The selected particles were combined in pairs and arranged into 20 groups according to the ratio of the particle size from small to large, and then the 20 groups of particles were subjected to particle packing tests. According to the basic model of particle packing (Equations (1) and (2)), the amount of coarse and fine aggregates is determined, and due to the wedging effect, there will be a residual number of particles in the actual packing process. The residual amount can be measured in the packing test, and the residual particle volume fraction is calculated as

(6) $$f_{1}\left(x_{i}\right)=\frac{{m_{di}}^{\prime }}{\rho_{i}\cdot V_{c}}$$

(7) $$f_{2}\left(x_{i}\right)=\frac{{m_{d_{i+1}}}^{\prime }}{\rho_{i+1}\cdot V_{c}}$$

where,$\;f_{1}\left(x_{i}\right)$—residual volume fraction of particles with particle size $d_{i}$, %;$\;f_{2}\left(x_{i}\right)$—residual volume fraction of particles with particle size$\;d_{i+1}$, %; ${m_{di}}^{\prime }$—residual mass of particles with particle size$\;d_{i}$, kg;$\;{m_{d_{i}+1}}^{\prime }$—residual mass of particles with particle size $d_{i+1}$, kg;$\;\rho_{i}—$apparent density of particles with particle size $d_{i}$, kg/m^3^;$\;\rho_{i+1}$—apparent density of particles with particle size $d_{i+1}$, kg/m^3^; $V_{c}$—container volume, m^3^.

The particle binary packing test and its results are shown in Table 1.

**Figure 1:**
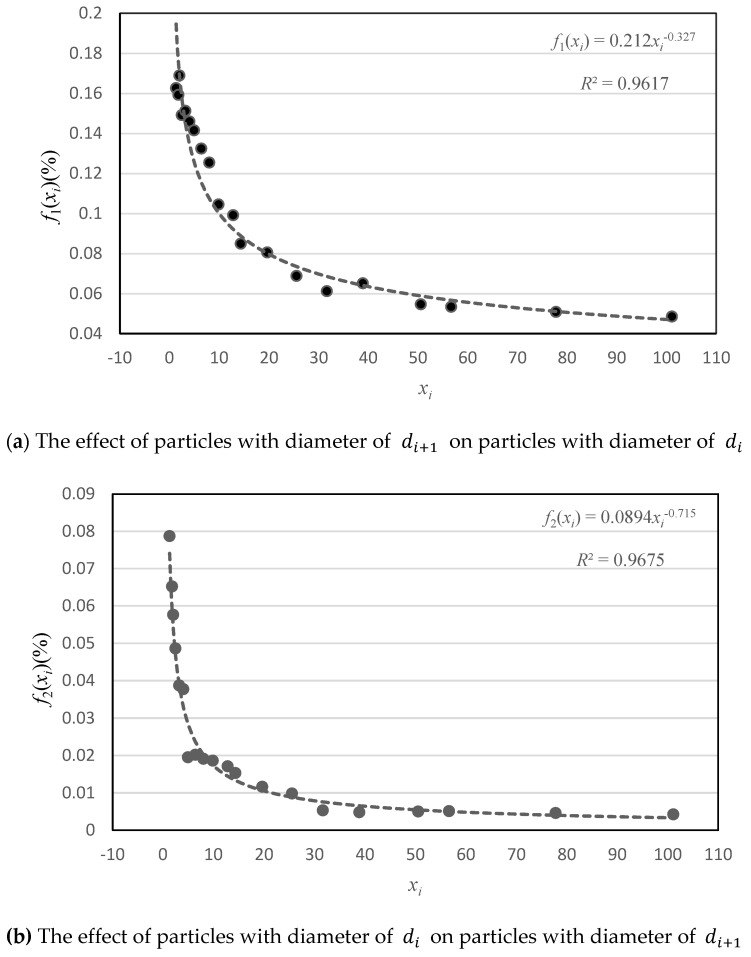
The relationship curve between the particle size ratio and the residual particle volume fraction.

The relationship curve between the particle size ratio and the residual particle volume fraction is fitted from the test results, as shown in Figure 1. It can be seen from Figure 1 that the particle size ratio *x_i_* had a similar effect on the residual particle volume fractions $f_{1}\left(x_{i}\right)$ and $f_{2}\left(x_{i}\right)$; that is, as *x_i_* increased, both $f_{1}\left(x_{i}\right)$ and $f_{2}\left(x_{i}\right)$ showed a downward trend. However, there was a difference in the degree of influence between particles of different sizes, i.e., when *x_i_* exceeded 5, the effect of particles with a diameter of $d_{i+1}$ on particles with a diameter of $d_{i}$ was significantly greater than that of particles with a diameter of $d_{i}$ on particles with a diameter of $d_{i+1}$.

#### 2.3.2. Basic Model Correction

In order to simplify the model, the following assumptions were made: (1) the concrete packing system was composed of different materials, and each material took its volume average particle size as its characteristic particle size; (2) there was a large difference in particle size between the concrete composition materials, such as coarse aggregate, fine aggregate, and cementitious material. We ignored the influence between non-adjacent size particles; that is, we only considered the effects between coarse aggregate and fine aggregate and the effects between fine aggregate and cementitious material, while the effects between coarse aggregate and cementitious material were ignored.

On the basis of the results of the particle packing test and the above assumptions, the basic model (Equations (1)–(4)) was modified, and the modified model expression is as follows:

Actual volume fraction of coarse aggregate:

(8) $${y_{1}}^{\prime }=1-{\varphi_{1}}^{\prime }$$

Actual volume fraction of fine aggregate:

(9) $${y_{2}}^{\prime }={\varphi_{1}}^{\prime }\cdot \left(1-{\varphi_{2}}^{\prime \prime }\right)$$

Actual volume fraction of cementitious material:

(10) $${y_{3}}^{\prime }={\varphi_{1}}^{\prime }\cdot {\varphi_{2}}^{\prime \prime }\cdot \left(1-{\varphi_{3}}^{\prime }\right)$$

Actual total volume fraction for particles packing:

(11) $$y^{\prime }=1-{\varphi_{1}}^{\prime }+{\varphi_{1}}^{\prime }\cdot \left(1-{\varphi_{2}}^{\prime \prime }\right)+{\varphi_{1}}^{\prime }\cdot {\varphi_{2}}^{\prime \prime }\cdot \left(1-{\varphi_{3}}^{\prime }\right)$$

where, ${\varphi_{1}}^{\prime },{\varphi_{2}}^{\prime \prime }\;\mathrm{and}\;{\varphi_{3}}^{\prime }$—actual voidage of coarse aggregate, fine aggregate, and cementitious materials.

Substituting the residual particle volume fractions into Equations (8)–(10), we get the following:

Actual volume fraction of coarse aggregate:

(12) $${y_{1}}^{\prime }=1-{\varphi_{1}}^{\prime }=\left(1-\varphi_{1}\right)\cdot \left(1-f_{1}\left(x_{1}\right)\right)$$

Actual volume fraction of fine aggregate:

(13) $${y_{2}}^{\prime }={\varphi_{1}}^{\prime }\cdot \left(1-{\varphi_{2}}^{\prime \prime }\right)=\varphi_{1}\cdot \left(1-\varphi_{2}\right)\cdot 1-f_{2}\left(x_{1}\right)\times \left(1-f_{1}\left(x_{2}\right)\right)$$

Actual volume fraction of cementitious material:

(14) $${y_{3}}^{\prime }={\varphi_{1}}^{\prime }\cdot {\varphi_{2}}^{\prime \prime }\cdot \left(1-{\varphi_{3}}^{\prime }\right)=\varphi_{1}\cdot \varphi_{2}\cdot \left(1-\varphi_{3}\right)\cdot \left(1-f_{2}\left(x_{2}\right)\right)$$

where, $\;f_{1}\left(x_{2}\right)\;\mathrm{and}\;f_{2}\left(x_{2}\right)$—the residual particle volume fractions of the particles with the size $d_{1}$ and $d_{2}$ when the size ratio is $x_{1}$;$\;f_{1}\left(x_{2}\right)\;\mathrm{and}\;f_{2}\left(x_{2}\right)$—the residual particle volume fractions of the particles with the size $d_{2}$ and $d_{3}$ when the size ratio is $x_{2}$.

#### 2.3.3. Solving the Actual Voidage of Particle Packing

Solved by Equations (12)–(14):

Coarse aggregate actual voidage:

(15) $${\varphi_{1}}^{\prime }=\varphi_{1}+f_{1}(x_{1})-\varphi_{1}\cdot f_{1}(x_{1})$$

Fine aggregate actual voidage:

(16) $${\varphi_{2}}^{\prime \prime }=1-\frac{\varphi_{1}\cdot \left[1+\varphi_{1}\cdot f_{2}(x_{1})-\varphi_{1}-f_{2}(x_{1})\right]\cdot \left[1-f_{2}(x_{1})\right]}{\varphi_{1}+f_{1}(x_{1})-\varphi_{1}\cdot f_{1}(x_{1})}$$

Actual voidage of cementitious material:

(17) $${\varphi_{3}}^{\prime }=1-\frac{(1-\varphi_{3})\cdot \left[1-f_{2}(x_{2})\right]}{1-f_{2}(x_{1})}$$

where,$\;f_{1}\left(x_{i}\right)=0.212x_{i}{}^{-0.327}$;$f_{2}\left(x_{i}\right)=0.0894x_{i}{}^{-0.715}$.

## 3. Concrete Mix Design

### 3.1. Materials

(1) Cement: ordinary Portland cement with strength grade 42.5; the main components are shown in Table 2, and the main technical properties are shown in Table 3.

(2) Fine aggregate: river sand in Zone II; the main technical properties are shown in Table 4.

(3) Coarse aggregate: crushed stone, 5–25 mm continuous gradation; the main technical properties are shown in Table 5.

(4) Water reducing agent: highly efficient polycarboxylic acid water reducing agent, light yellow liquid with density of 1.1 g/cm^3^ and pH of 7.0–8.0.

(5) Water: drinking water.

### 3.2. Actual Particle Voidage

On the basis of the main technical parameters of the concrete constituent materials, the actual voidages of the granular material in the concrete were calculated from Equations (15)–(17), as shown in Table 6.

It can be seen from Table 6 that under consideration of the interaction between particles in the packing system, the actual void ratio between the particles increased; that is, the coarse aggregate voidage increased from 46.2% to 50.8%, the fine aggregate voidage increased from 43.5% to 49.0%, and the cement voidage increased from 52.5 to 55.6%.

### 3.3. Concrete Composition Materials per m^3^

Three water–binder ratios of 0.42, 0.47, and 0.52 were selected, and the design slump of the concrete mixture was 70–90 mm.

The calculation process of the amount of crushed stone, sand, cement, and water in 1 m^3^ concrete is as follows:

(18) $$m_{1}=V\cdot {y_{1}}^{\prime }\cdot \xi \cdot \rho_{01}$$

(19) $$m_{2}=V\cdot {y_{2}}^{\prime }\cdot \xi \cdot \rho_{02}$$

(20) $$m_{3}=V\cdot {y_{3}}^{\prime }\cdot \xi \cdot \rho_{03}$$

(21) $$m_{w}=V\cdot y_{w}\cdot \xi \cdot \rho_{w}$$

where $m_{1},\;m_{2},\;\mathrm{and}\;m_{w}$—mass of crushed stone, sand, cement, and water, kg;$\;\rho_{01},\;\rho_{02},\;\mathrm{and}\;\rho_{w}$—apparent density of crushed stone, sand, cement, and water, kg/m^3^; *V*—concrete volume, taken as 1 m^3^; *ξ*—the volume shrinkage coefficient is expressed as follows:

(22) $$\xi =\frac{1}{{y_{1}}^{\prime }+{y_{2}}^{\prime }+{y_{3}}^{\prime }+y_{w}}$$

where *y*_w_ is the volume fraction of water, expressed as follows:

(23) $$y_{w}=\frac{W}{B}\cdot \frac{\rho_{03}\cdot {y_{3}}^{\prime }}{\rho_{w}}$$

where *W*/*B* is the water–binder ratio.

Put Equations (8)–(10), (22), and (23) and various material parameters into Equations (18)–(21), and you can get the amount of crushed stone, sand, cement, and water in 1 m^3^ concrete, see C-1, C-2, and C-3 in Table 7.

In Table 7, C0-1, C0-2, and C0-3 are the control concrete, and the mix ratios design are based on *Specification for Mix Proportion Design of Ordinary Concrete* (National standard of the People’s Republic of China, JGJ55-2011). The amount of the water reducing agent given in Table 7 was determined by the workability test of the concrete mixture. The concrete mixes all met the designed slump of 70–90 mm, and there was no demixing or segregating.

## 4. Compressive Strength Test of Concrete

**Figure 2:**
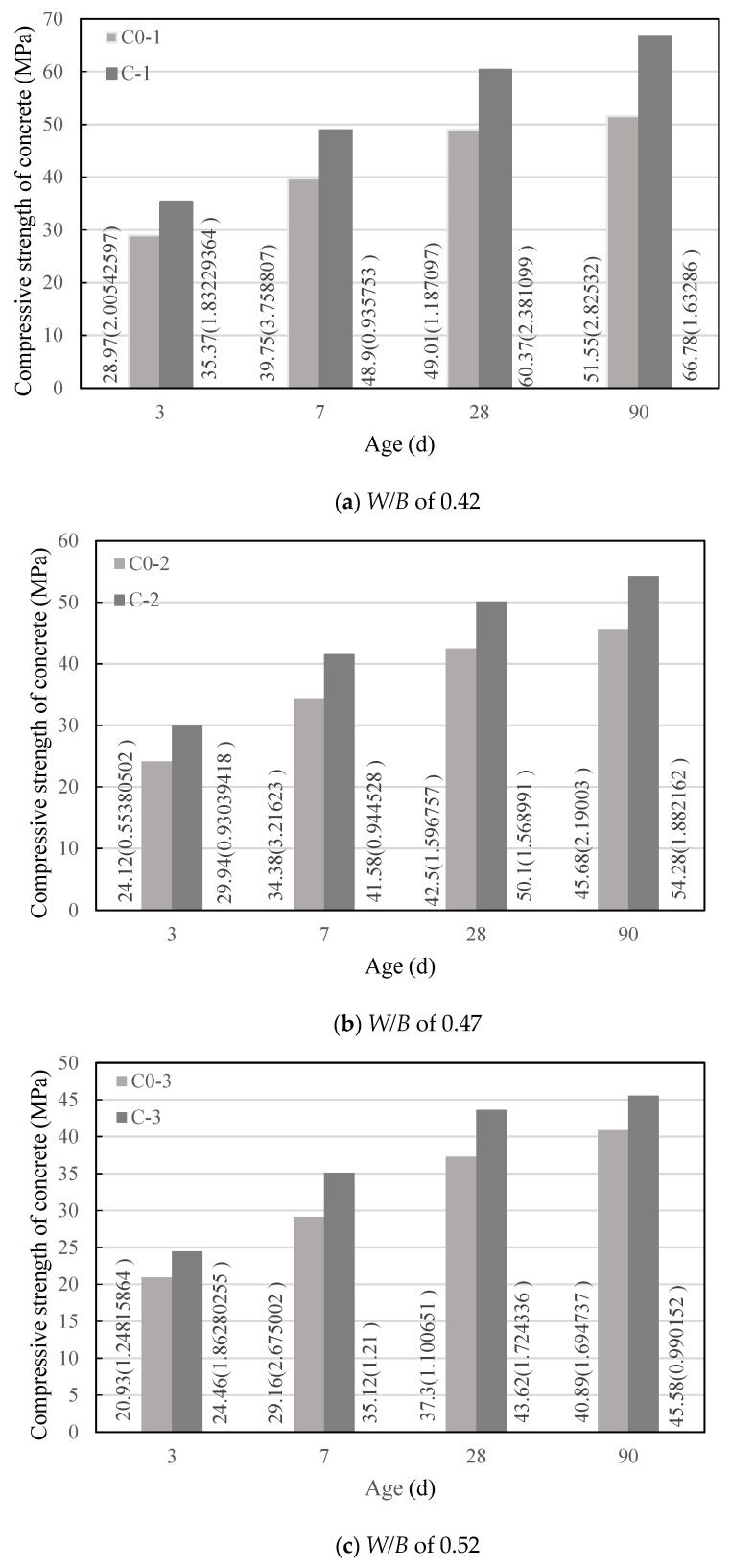
Compressive strength of concrete.

The test was performed in accordance with the concrete compressive strength test method specified in *Standard for Test Method of Mechanical Properties on Ordinary Concrete* (National standard of the People’s Republic of China, GB/T50081-2002). In this method, there are three test pieces with a size of 100 mm × 100 mm × 100 mm in each group, and the test result is the average of the compressive strength test values of the three test pieces. The test results are shown in Figure 2, in which the data beside the column are the average value and standard deviation (in parentheses) of the compressive strength, and the experimental data are less discrete.

It can be seen from Figure 2 that under water–binder ratios of 0.42, 0.47, and 0.52, the development law of the compressive strength of concrete in group C with age was basically consistent with the development law of the compressive strength of control concrete (C0) with age, and the compressive strength of concrete in both groups increased with the extension of the age. At the same age, the changing law of compressive strength of concrete in group C with the water–binder ratio was the same as the changing law of compressive strength of concrete in group C0 with the water–cement ratio. The compressive strength of concrete in both groups decreased with the increase in water–cement ratio; however, when the water–binder ratio was 0.42, 0.47, or 0.52, the compressive strength of concrete in group C was higher than that of the control concrete with the same water–binder ratio. The growth rate of the compressive strength of concrete is shown in Table 8. It can be seen from Table 8 that the concrete with a water–binder ratio of 0.42 had the highest growth rate of compressive strength at each age, followed by the concrete with a water–binder ratio of 0.47 and then 0.52. Except for a single point, it can be said that the smaller the water–binder ratio, the greater the growth rate of compressive strength at each age.

## 5. Elastic Modulus Test of Concrete under Static Compression

**Figure 3:**
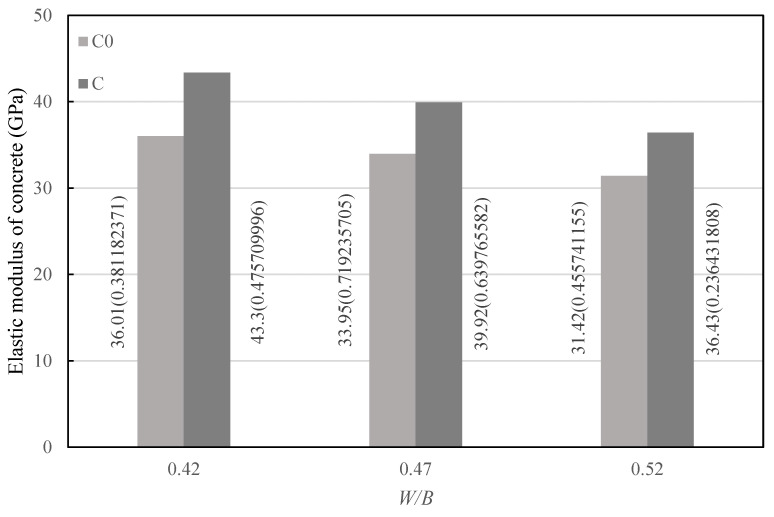
Elastic modulus of concrete.

The test was carried out according to the test method of elastic modulus of concrete under static compression specified in *Standard for Test Method of Mechanical Properties on Ordinary Concrete* (National standard of the People’s Republic of China, GB/T50081-2002). In the method, each group had six specimens with the size of 150 mm × 150 mm × 300 mm, which were cured for 28 d before testing. Among them, three specimens were used to test the axial compressive strength of concrete, and the other three specimens were used to test the elastic modulus of concrete. The test results are taken as the average value of the tested elastic modulus of the three specimens, as shown in Figure 3, in which the data beside the column are the average value and standard deviation (in parentheses) of the elastic modulus, and the experimental data are less discrete.

As can be seen from Figure 3, under a water–binder ratio of 0.42, 0.47, and 0.52, the variation law of the elastic modulus of concrete in group C with the water–binder ratio was basically consistent with the variation law of the elastic modulus of control concrete with the water–binder ratio. When the water–binder ratio was 0.42, 0.47, or 0.52, the elastic modulus of concrete in group C was higher than that in group C0 with the same water–cement ratio. Specifically, when the water–binder ratio was 0.42, the elastic modulus of concrete increased by 20.44%; when the water–binder ratio was 0.47, the elastic modulus of concrete increased by 17.55%; when the water–binder ratio was 0.52, the elastic modulus of concrete increased by 15.95%. The smaller the water–binder ratio, the greater the degree of increase in concrete elastic modulus.

**Figure 4:**
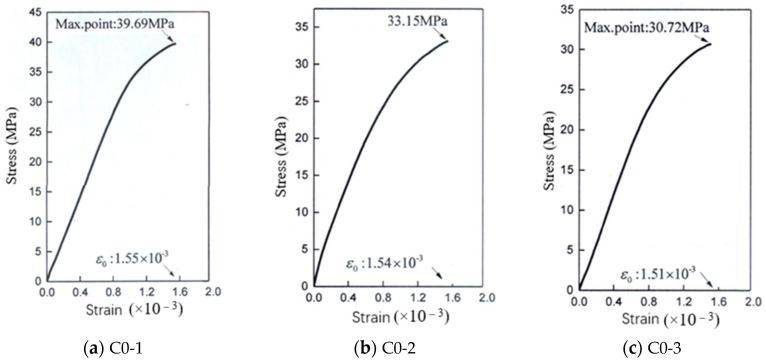

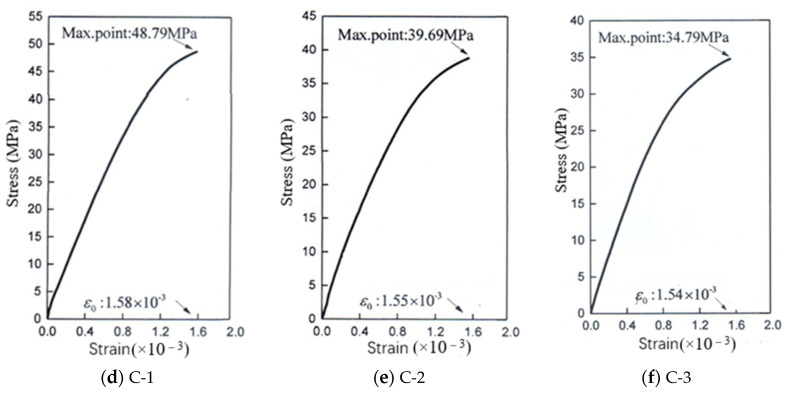
The relationship between stress and strain of concrete.

The relationship between stress and strain before the ultimate stress of the concrete under axial compression is shown in Figure 4. It can be seen from Figure 4 that under the same water–cement ratio, the rising section of the stress–strain curves of group C was steeper than that of group C0, and the ultimate stress increased.

## 6. Analysis and Discussion

The concrete mix design method proposed in this study was based on dense particle packing. Since the aggregate was a granular material with the largest proportion in concrete, the volume fraction of aggregate in the concrete prepared by this method increased to different degrees. The volume fraction of aggregate is shown in Table 9. As can be seen from Table 9, when the water–binder ratio was 0.42, 0.47, and 0.52, the aggregate volume fraction increased by 18.72%, 13.49%, and 8.97%, respectively.

**Figure 5:**
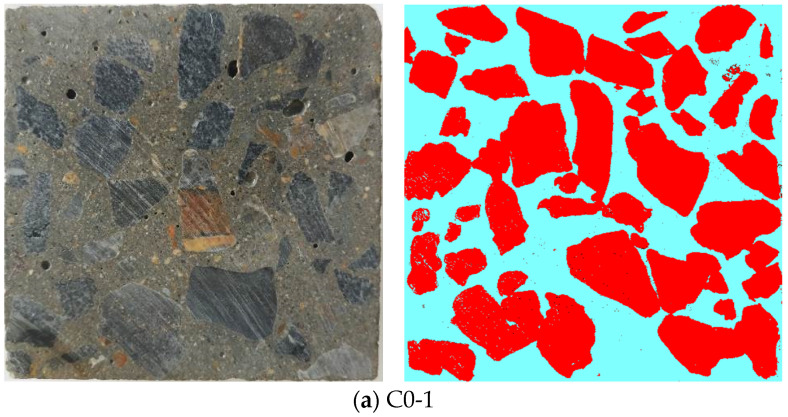

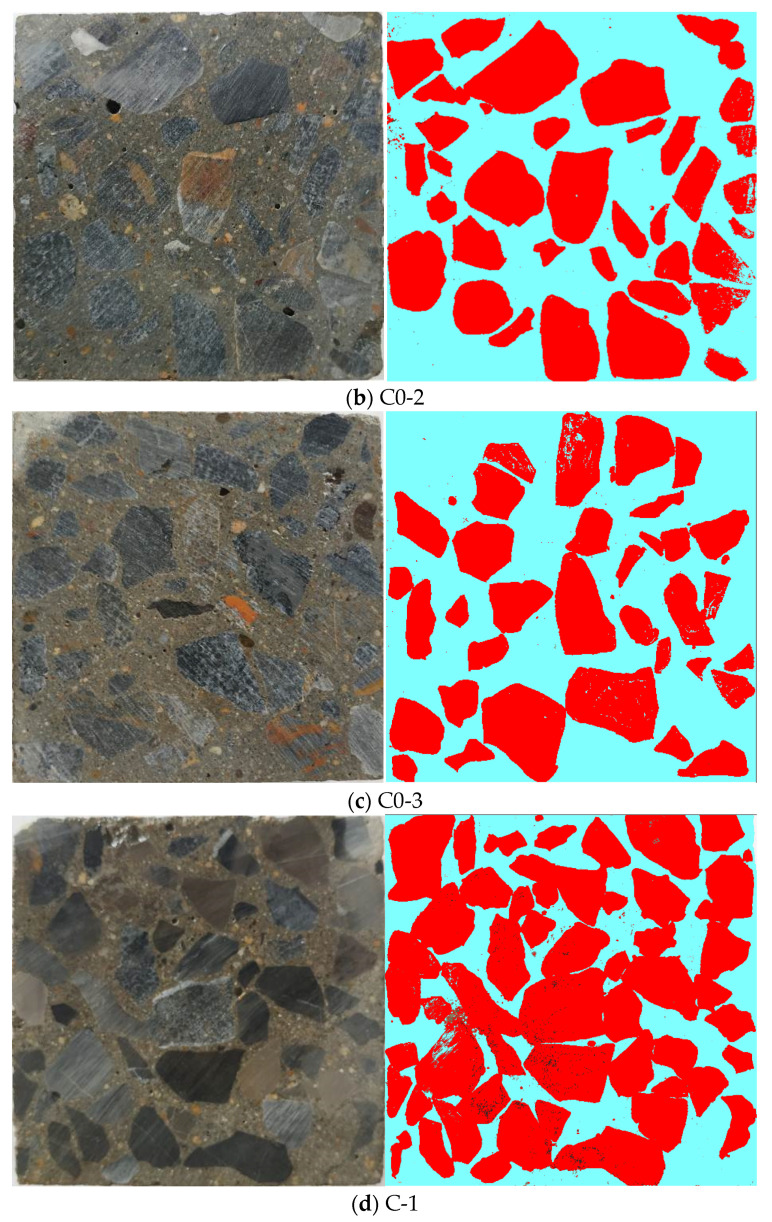

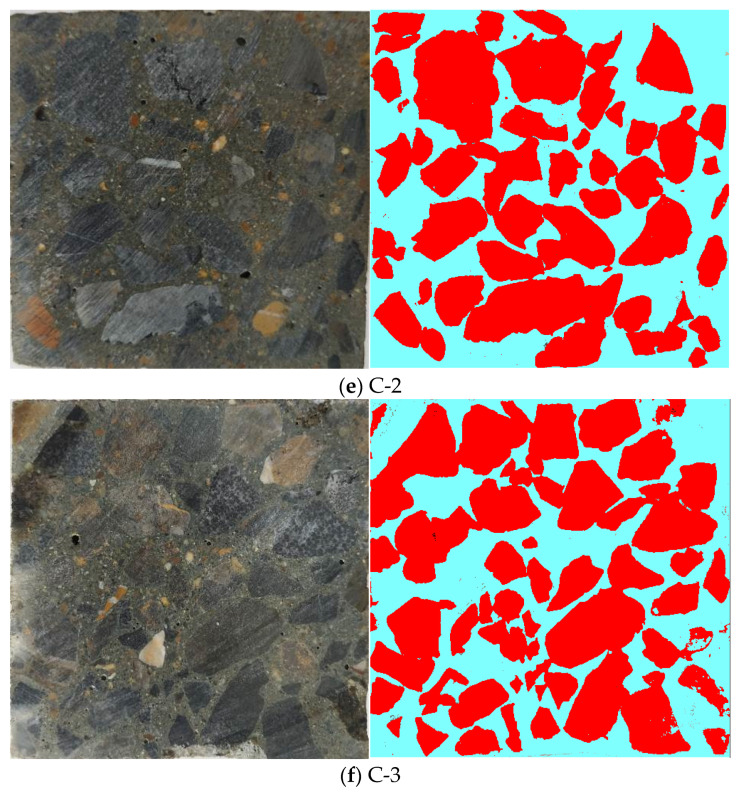
Distribution of coarse aggregate in sections of concrete.

Sections of the concrete specimen were randomly taken. The coarse aggregate information was extracted on the section using the IPP image processing software, and grayscale processing was performed, as shown in Figure 5. From Figure 5, the distribution of coarse aggregate in concrete can be intuitively seen, among which the coarse aggregate in group C0 was suspended in the cement mortar matrix, while the coarse aggregate in group C was closely arranged and even overlapped with each other, forming a relatively dense concrete core skeleton.

Concrete is a multiphase composite material composed of mortar, coarse aggregate, and an interface transition zone between the two. Since aggregate has a large volume fraction in concrete, especially coarse aggregate, it has a great influence on mechanical parameters such as compressive strength and elastic modulus under the conditions of a certain cementitious material and water–cement ratio [21].

In concrete of group C, the higher aggregate volume fraction makes the aggregate more closely distributed in the concrete (as can be seen from Figure 5), so that the aggregate acts as a larger skeleton in the concrete. In addition, it can be seen from Table 8 that when the water–binder ratio was 0.42, 0.47, and 0.52, the increase degree in the compressive strength of the concrete in group C increased with the decrease in the water–binder ratio. This is mainly because the increase rate in the aggregate volume fraction of the concrete in group C increased as the water–binder ratio decreased (see Table 9), which also proves from another perspective that the increase in aggregate volume fraction is beneficial to the improvement of the compressive strength of concrete. In this study, the compressive strength of concrete increased with the increase in aggregate volume fraction, which is consistent with the research results in reference [22].

**Figure 6:**
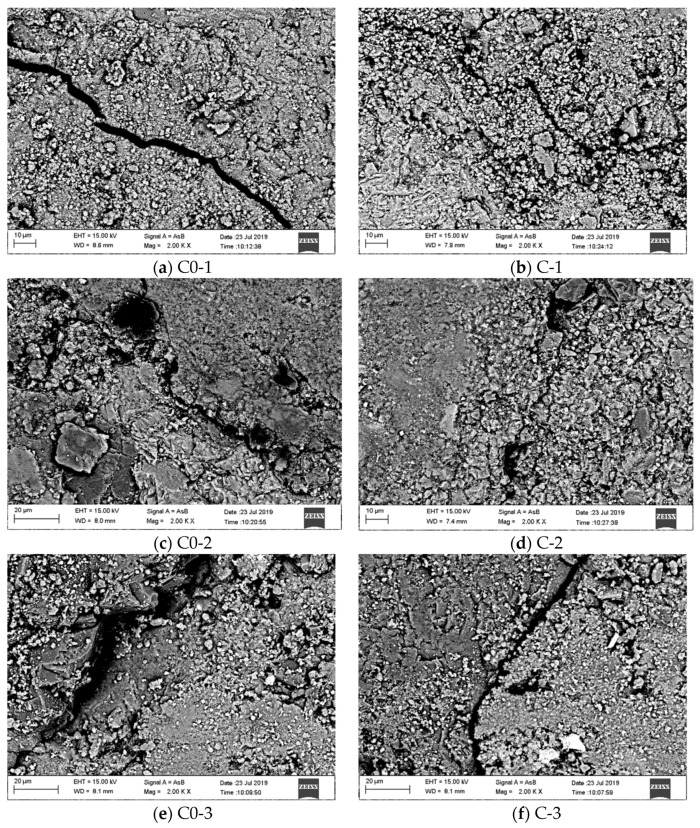
Micro-morphology of the interface transition zone in the concrete (×2000).

Figure 6 shows the SEM micro-morphology of the concrete interface transition zone. It can be seen from Figure 6 that under water–binder ratios of 0.42, 0.47, and 0.52, there were obvious micro-cracks and defects in the interface transition zone of the control concrete; while the micro-cracks in the interface transition zone of the concrete in group C were not as obvious as those in the control concrete with the same water–binder ratio, and the defects were also relatively few. The micro-cracks in the interface transition zone are mainly due to the early shrinkage of concrete, and the increase in aggregate volume fraction will increase the degree of restraint of the aggregate on the shrinkage of the slurry, thereby improving the micro-cracks in the interface transition zone [23]. The compressive failure of ordinary concrete mainly occurs in the interface transition zone and the cement stone part, so the improvement of the interface transition zone will directly lead to the increase in concrete compressive strength.

It can be seen from Figure 3 that when the water–binder ratio was 0.42, 0.47, or 0.52, the elastic modulus of concrete in group C was higher than that of control concrete with the same water–binder ratio, and the increase degree of the elastic modulus also increased as the water–binder ratio decreased. Aggregate is the component with the largest elastic modulus in concrete, so the increase in aggregate volume fraction can increase the elastic modulus of concrete to a certain extent. As can be seen from Figure 4, when the water–binder ratio was 0.42, 0.47 or 0.52, the ultimate stress of concrete in group C was greater than that of control concrete with the same water–binder ratio under axial compression, indicating that the elastic modulus of concrete has a positive correlation with the compressive strength, which is consistent with the research results in the literature [22].

In addition, it can be seen from Table 7 that when the water–binder ratio was 0.42, 0.47, or 0.52, the amount of cement in group C was lower than that in group C0 with the same water–binder ratio. Portland cement production requires high amounts of energy, releasing a considerable amount of CO_2_ [16], and a large amount of energy consumption and CO_2_ emissions will have a great impact on the human living environment. Therefore, reducing the amount of cement in concrete has greater environmental protection and sustainable development significance.

## 7. Conclusions

This research proposed a concrete mix design method based on the principle of dense packing of particles, and the method was used to prepare concrete. Compressive strength and elastic modulus of the concrete were experimentally studied. The coarse aggregate distribution and interface transition zone in the concrete were observed, and the following conclusions were drawn:

(1) The design method of concrete mix proportion based on interparticle voidage proposed in the research is a kind of design method for concrete mix proportion according to the principle of dense particle packing.
(2) The functional relationship between the particle size ratio and the remaining volume fraction of the particles is the basis for determining the actual voidage of the granular material in the concrete.
(3) The coarse aggregates in the concrete prepared by the method proposed in this research are closely distributed, and even overlap each other, forming a relatively dense concrete core skeleton. Micro-cracks and defects in the interface transition zone of the concrete are also reduced to a certain extent.
(4) In the concrete, there is an increase in aggregate volume fraction and a corresponding decrease in the amount of cement, and when an appropriate amount of water reducing agent is added, the workability of the concrete mixture meets the design requirements.
(5) When the water–binder ratio was 0.42, 0.47, or 0.52, the compressive strength of concrete with ages of 3, 7, 28, or 90 d all increased compared with that of the control concrete at the same age, and the degree of increase in compressive strength increased as the water–binder ratio decreased.
(6) When the water–binder ratio was 0.42, 0.47, or 0.52, the static elastic modulus of concrete increased compared with that of the control concrete, and the degree of increase in static elastic modulus increased as the water–binder ratio decreased, showing that the elastic modulus is positively correlated with the compressive strength.

## Figures and Tables

**Table 1:** The particle binary packing test.

No.	$x_{i}$	$\mathrm{Particle}\;\mathrm{Size}\;\mathrm{Composition}\;\mathrm{of}\;d_{i}\;and\;d_{i+1}$ $\left(d_{i}+d_{i+1}\right)$	$m_{d_{i}}\prime (\mathrm{kg})$	$m_{d_{i}+1}\prime (\mathrm{kg})$	$f_{1}\left(x_{i}\right)$	$f_{2}\left(x_{i}\right)$
1	1.3	22.75 + 17.5	4.434	2.146	0.1626	0.0787
2	1.784	22.75 + 12.75	4.341	1.778	0.1592	0.0652
3	2	0.45 + 0.225	4.403	1.502	0.1689	0.0576
4	2.456	17.5 + 7.125	4.066	1.325	0.1491	0.0486
5	3.193	22.75 + 7.125	4.123	1.055	0.1512	0.0387
6	4.01	7.125 + 1.777	3.979	0.983	0.1459	0.0377
7	4.923	17.5 + 3.555	3.859	0.508	0.1415	0.0195
8	6.4	22.75 + 3.555	3.611	0.527	0.1324	0.0202
9	8.006	7.125 + 0.89	3.420	0.498	0.1254	0.0191
10	9.848	17.5 + 1.777	2.850	0.485	0.1045	0.0186
11	12.8	22.75 + 1.777	2.702	0.446	0.0991	0.0171
12	14.326	12.75 + 0.89	2.315	0.399	0.0849	0.0153
13	19.663	17.5 + 0.89	2.195	0.302	0.0805	0.0116
14	25.56	22.75 + 0.89	1.876	0.255	0.0688	0.0098
15	31.667	7.125 + 0.225	1.669	0.138	0.0612	0.0053
16	38.889	17.5 + 0.45	1.775	0.125	0.0651	0.0048
17	50.556	22.75 + 0.45	1.489	0.130	0.0546	0.0050
18	56.667	12.75 + 0.225	1.456	0.133	0.0534	0.0051
19	77.778	17.5 + 0.225	1.385	0.120	0.0508	0.0046
20	101.111	22.75 + 0.225	1.323	0.109	0.0485	0.0042

Note: *x_i_* = *d_i_*/*d_i_*_+1_.

**Table 2:** Main components of cement (mass fraction).

Main Mineral Composition(%)				Main Chemical Composition(%)						
C_2_S	C_3_S	C_3_A	C_4_AF	SiO_2_	Al_2_O_3_	Fe_2_O_3_	CaO	MgO	SO_3_	LOI
21.8	51	11	13	20.18	4.98	3.28	60.92	4.59	1.78	3.48

**Table 3:** Main technical properties of cement.

Strength Grade	Setting Time (min)		Apparent Density (g/cm^3^)	Compressive Strength (MPa)		Soundness	Volume Average Particle Size (μm)	Voidage (%)
	Initial	Final		3d	28d			
42.5	176	403	3.158	22.6	49.8	Qualified	20.8	52.5%

**Table 4:** Main technical properties of sand.

Apparent Density (kg/m^3^)	Bulk Density (kg/m^3^)	Voidage(%)	Volume Average Particle Size (mm)	Fineness Modulus
2607	1473	43.5	0.99137	2.51

**Table 5:** Main technical properties of crushed stone.

Apparent Density (kg/m^3^)	Bulk Density (kg/m^3^)	Voidage (%)	Volume Average Particle Size (mm)	Crushing Index (%)
2727	1466	46.2	15.862	9

**Table 6:** Actual voidages of granular materials in concrete.

	Volume Average Particle Size (mm)	$x_{i}$	$f_{1}(x_{i})$	$f_{2}(x_{i})$	$\varphi_{i}(\%)$	Actual Voidage (%)
Coarse aggregate	15.862	16	0.0856	0.0123	46.2	50.8
Fine aggregate	0.99137	47.662	0.06	0.00565	43.5	49.0
Cement	0.0208	1.359	0.192	0.0817	52.5	55.6

**Table 7:** Concrete mix ratio.

No.	*W/B*	Cement (kgm^3^)	Sand (kgm^3^)	Crushed Stone (kgm^3^)	Water (kgm^3^)	Water Reducer Mass Fraction (Percentage of Cement Mass) (%)
C0-1	0.42	511.9	511.0	1137.4	215.0	0
C0-2	0.47	457.5	558.7	1134.1	215.0	0
C0-3	0.52	413.5	605.1	1123.7	215.0	0
C-1	0.42	369.2	626.1	1330.0	155.1	0.6
C-2	0.47	362.7	615.1	1306.7	170.5	0.4
C-3	0.52	356.1	603.9	1282.9	185.2	0.2

**Table 8:** Growth rate of compressive strength of concrete (%).

No.	Age (d)			
	3	7	28	90
C-1	22.09	23.02	23.18	29.54
C-2	24.13	20.94	17.88	18.80
C-3	16.87	20.44	16.94	11.47

**Table 9:** Aggregate volume fraction in concrete.

	C0-1	C0-2	C0-3	C-1	C-2	C-3
Aggregate volume fraction (%)	61.31	63.02	64.42	72.79	71.52	70.20
Increase rate of aggregate volume fraction (%)	—	—	—	18.72	13.49	8.97

## Data Availability

The data presented in this study are openly available.
